# Post-COVID-19 metabolic syndrome: a new challenge for nursing care*

**DOI:** 10.17533/udea.iee.v41n1e01

**Published:** 2023-03-14

**Authors:** João Cruz, Tahissa Frota Cavalcante, Nuno Damácio de Carvalho Félix

**Affiliations:** 1 Nurse, Master’s Student in Nursing. University for International Integration of the Afro-Brazilian Lusophony, Redenção (Brazil). E-mail: enfjcncruz@gmail.com. https://orcid.org/0000-0002-0972-2988 University for International Integration of the Afro-Brazilian Lusophony Redenção Brazil enfjcncruz@gmail.com; 2 Nurse, PhD. Assistant Professor, University for International Integration of the Afro-Brazilian Lusophony, Redenção (Brazil). https://orcid.org/0000-0002-2594-2323 E-mail: tahissa@unilab.edu.br University for International Integration of the Afro-Brazilian Lusophony Redenção Brazil; 3 Nurse, PhD. Assistant Professor, Federal University of Recôncavo da Bahia, Santo Antônio de Jesus (Brazil). https://orcid.org/0000-0002-0102-3023 E-mail: nunofelix@ufrb.edu.br Universidade Federal do Recôncavo da Bahia Federal University of Recôncavo da Bahia Santo Antônio de Jesus Brazil nunofelix@ufrb.edu.br

In crisis scenarios, the professions that have the essence of caring for are highlighted. In this sense, during the COVID-19 pandemic, nursing played a key role in the identification, testing, care and rehabilitation of patients.^1^ It was through this profession that many clients were fully assisted in the process of illness/rehabilitation, revealing the importance of the category for the health system; a fact that also persists in a post-COVID scenario. COVID-19 led to the collapse of global health with high rates of mortality and hospital morbidity, generating an estimated 18.2 million deaths around the world.^2^ Nevertheless, it is known that COVID-19 is a respiratory disease with vascular implications that interact with cardiometabolic factors such as oxidative stress, endothelial dysfunction, insulin resistance, atherosclerosis, overweight, increased body fat and alterations in the microbiome, leading to systemic complications and death.^3^

The vascular manifestations of COVID-19 include severe myocardial injury, heart failure, endothelial cell injury, arrhythmias, brain injury and hyper-reactive inflammation, leading to thrombosis, which trigger changes in the metabolic patterns of individuals and interfere with the risk factors for both diseases.^4^^,^^5^ That is why patients infected with COVID-19 have a thirteen-fold increased risk of hypertension, cardio- and cerebrovascular diseases and diabetes.^6^ It is noteworthy, therefore, that the metabolic alterations related to COVID-19 reveal the imminence of a new health hazard, the post-COVID metabolic syndrome. In the context of nursing, the metabolic syndrome is understood as the aggregation of cardiovascular and multifactorial risk markers, with asymptomatic inflammation that predisposes to vulnerability.^7^

With the increase in COVID-19 cases in the world, primary factors and alterations of cardiometabolic metabolism have become a public health problem. From the interaction between the two diseases, studies already point to the emergence of a metabolic syndrome by COVID-19, which in its early stage entails the worst clinical prognosis in COVID-19, including hypercytokinemia, inflammation, severe acute respiratory syndrome, abdominal adiposity, cardiovascular disease, hypercoagulability and hydroelectrolyte imbalances.^8^^,^^9^ Multiple organ failure is among the main complications of the metabolic syndrome from COVID-19, which, in addition to mortality during hospitalization, increases the chances of disability for patients who manage to recover partially.^10^^-^^11^ That is why studies in this theme try to elucidate strategies to control the disease, understand the symptoms and the involved pathophysiology, detect the risk of aggravation in potentially fatal patients and stabilize cardiometabolic risk factors.^12^ On the other hand, this has been an important gap in the literature.^9^

In the nursing field, research related to COVID-19 in primary care has revealed fear regarding the clinical repercussions of the disease, linked to the strong thought of personal/professional fragility.^13^ On the other hand, it is reinforced the need to better understand the mechanism of infection, to combat the high levels of stress and fatigue due to the exhausting workload in hospitals.^14^ In addition, much has been discussed in nursing research about the mental health of professionals, shortage or usability problems in personal protective equipment and psychosomatic disorders.^15^ In the management field, it is observed that there is a need to identify the impacts of the pandemic scenario, assigning value on the solvability of nursing actions^16^ and changes in paradigms regarding the metabolic syndrome disorders by COVID-19.

In the long COVID-19, the nursing work is based on the implementation of strategies that make supported care possible by increasing the level of education of professionals, strengthening the support network and implementing assistive technologies such as telecommunication and the use of robotics.^17^ The challenge of nursing care is not only in the nurse-patient field, but also in the care for and adaptation to oneself. As it is seen sometimes as the angel or hero of care, the nursing team deserves, consequently, the visibility necessary to achieve an effective recruitment of new nurses in view of the eminent evasion of services and the appearance of cardiometabolic factors.^18^ Given the epidemiological presence of a clinical syndrome with global alert, it is necessary to call attention to international coordination and collaboration, these measures being essential for public health and implying the convocation of nurses in the management of this scenario. In this sense, strategies for management, health education, as well as advanced nursing practices with moderate interventions conducted by nurses, will be necessary for effective health care.

Expanding people’s access to laboratory testing after contracting COVID-19 is an effective screening strategy.^8^ In the collective health field, the active search for patients who had COVID-19 and present the comorbidities that can lead to clinical worsening and the syndrome between the two diseases is pertinent. In this space, it is considered essential the performance of the nursing team in holistic health care, leading self-care practices. It is also up to nursing to lead the strategy against the increase in cases of both diseases and clinical worsening. The epidemiological scenario is then a challenge for nursing practices; however, it reveals itself as an opportunity for empowerment of care practices, as well as social and scientific responsibility, in order to mitigate the cases of this syndrome. Especially in pandemic disease, it is important to identify culturally adapted care observing the specific realities of the population and their social and health contexts with meta-paradigmatic perspectives to guide professional work with a focus on environment, person, health and nursing care. 

Finally, it is emphasized the need for continuous training of health teams, especially the nursing team, in the proactive engagement of health work, reinforcing prevention and promotion practices, providing critical and scientific tools in social and cultural contexts with care plans focused on control, monitoring and evaluation, with a view to achieving feasible indicators of nursing practice.
